# Alcohol-based microextraction of Rhodamine 6G from soft drink, food, and cosmetic samples

**DOI:** 10.55730/1300-0527.3799

**Published:** 2026-04-13

**Authors:** Mustafa SOYLAK, Esra YAMANER, Özgür ÖZALP

**Affiliations:** 1 Department of Chemistry, Faculty of Sciences, Erciyes University, Kayseri, Turkiye; 2 Technology Research and Application Center, Erciyes University, Kayseri, Turkiye; 3 Turkish Academy of Sciences, Ankara, Turkiye

**Keywords:** Rhodamine 6G, UV-Vis spectrophotometer, alcohol-based solvent extraction, high-performance liquid chromatography with fluorescence detection, cosmetics and food products

## Abstract

By using alcohol-based solvent extraction without tetrahydrofuran, a liquid–liquid microextraction technique was developed for the separation–enrichment of trace levels of Rhodamine 6G from complex matrices, including soft drinks, food, cosmetics, hygiene products, and natural waters. Numerous experimental parameters were tuned, including pH, alcohol volume, ultrasonication time, and sample and eluent volume. The enrichment factor (EF), limit of detection (LOD), and limit of quantification (LOQ) were used to assess the analytical performance of the suggested approach. The LOD and LOQ were 6.92 ng mL^−1^ and 23 ng mL^−1^, respectively, and the EF was 14. Through the use of the standard addition method, the proposed alcohol-based solvent extraction method was effectively employed to determine trace amounts of Rhodamine 6G, particularly in soft drinks, cosmetics, hygiene products, food, and natural waterways. High-performance liquid chromatography with fluorescence detection was also used to validate the method in real samples. The suggested method offers a more straightforward extraction process, less solvent consumption, and quick analyte recovery when compared to previously published extraction techniques, underscoring its potential as a useful and ecofriendly substitute for Rhodamine 6G determination.

## 1. Introduction

The increasing global population and industrialization have directly led to an increase in food and environmental pollution. Wastewater from dyes has significant impacts on human health and the natural environment. Some dyes can be hazardous when dissolved in water, even in small amounts [1,2]. Rhodamine 6G can generate major pollutants in water and ecosystems when mixed with water from various industrial wastes [3]. Dyes include complicated aromatic molecular structures. Anionic acid dyes, cationic basic dyes, and nonionic dyes are classified based on their solubility in aqueous solutions [4]. Importantly, cationic dyes are considered more poisonous than anionic dyes and most synthetic dyes are carcinogenic. When considering production procedures, Rhodamine 6G is a cationic dye with a more hazardous structure than other related dyes [5]. Rhodamine 6G, among the oldest synthetic dyes, is a derivative of xanthan dyes, which are highly soluble in water. Rhodamine 6G is used as a colorant in the textile, paper, plastic, paint, cosmetics, and food industries due to its low cost, bright pink color, and fluorescent properties [6,7]. Rhodamine 6G is often used to improve visual aesthetics and increase sales. It is also illegally added to food products. When released into the environment, it harms both the environment and living organisms [8]. The use of Rhodamine 6G as a food dye has raised serious concerns because it causes diseases such as asthma and allergies, as well as skin, eye, and respiratory irritation; cancer; reproductive problems; and chronic toxicity in living organisms [9–11]. In 1983, the Food and Drug Administration declared that numerous dyestuffs were carcinogenic based on testing and prohibited their use globally [12]. Nonetheless, some producers continue to add trace amounts of these unlawful dyestuffs to related items, endangering the health of living beings. Rhodamine 6G cannot be determined directly using laboratory instruments; expensive technologies are necessary [13,14].

In line with green chemistry principles, priority has been given to the use of low-toxicity solvents, reduction of waste, energy efficiency, and operational safety criteria. The method developed provides fast, effective, and analytically accurate extraction using low volumes of solvent. In this context, Rhodamine 6G extraction using 1-decanol as extraction solvent offers a robust and feasible alternative in terms of analytical performance, environmental sustainability, and human health.

Due to their practical usage during the determination of dyes, spectrophotometric techniques are an excellent choice with broad usability, including measurements in the ultraviolet and visible spectrophotometer as the most convenient, low-cost, accessible equipment [15–17]. Therefore, the UV–visible spectrophotometer is utilized in every laboratory due to its easy access and fast response without requiring an expert^1^ [18]. Although the use of spectrophotometer-like devices is aimed at analyzing dyes in food products, no analysis technique is sufficient for the analysis of food dyes at trace levels [19]. Due to the matrix effect and low detection limits, it is necessary to develop an additional method. Among these methods, extraction techniques such as separation–enrichment methods [20] are among the most frequently used by researchers in terms of both cost and simplicity. Among the extraction techniques, liquid–liquid extraction (LLE) [21–23], cloud point extraction (CPE) [24,25], dispersive liquid–liquid microextraction (DLLME) [26,27], ultrasound-assisted ionic liquid dispersive liquid–liquid phase microextraction (UA-IL-DLLLME) [28–30], liquid phase microextraction (LPME) [31,32], solid phase microextraction (SPME) [33,34], and alcohol-based solvent extraction [35] enable the detection of very low levels of analytes and prevent interference issues.

Among these advantages, liquid–liquid extraction stands out due to its use of nontoxic solvents and its low cost. Deep eutectic solvents^2^ [36,37], supramolecular solvents, switchable (exchangeable) solvents [38], ionic liquid solvents [39] and alcohol-based solvent extraction [40] are utilized for separation.

Alcohol-based solvent extraction provides a suitable medium for the separation and enrichment of analytes due to their wide polarity range, since they provide various interactions (hydrophobic, hydrogen bonding, electrostatic, π–cation, etc.) with target analytes [41,42]. Alcohol-based solvent extraction retains their parent molecules by noncovalent interactions such π–π stacking, van der Waals forces, and hydrogen bonding. Moreover, alcohol-based solvent extraction results in a large number of analyte binding sites. This attribute provides great extraction efficiency due to low extraction volumes. These qualities have increased the development potential for sample preparation in the testing of food, environmental, and organic products [43–46].

1-Decanol (CH_3_(CH_2_)_9_OH), a 10-carbon, linear-chain primary alcohol, is used as an extraction solvent. The long alkyl chain in its structure confers hydrophobic properties, while the hydroxyl group provides partial hydrophilicity. This dual-character structure enables the efficient transport of various analytes into the solvent phase. Its high boiling point (230 °C) and low vapor pressure (1 hPa at 20 °C) ensure a safe working environment in laboratory applications. 1-Decanol is widely utilized in microextraction techniques due to its advantages such as low solvent consumption, high enrichment factor, cost-effectiveness, and the ability to prepare solvent phases with different polarities. Additionally, its biodegradable structure makes it an important alternative in terms of environmental sustainability. Its compatibility with common analytical techniques such as UV–Vis spectrophotometry and high-performance liquid chromatography with fluorescence detection (HPLC-FLD) facilitates postextraction analysis processes [47–50]. The determination of Rhodamine 6G using sensitive, selective, and environmentally friendly methods is of great importance. The 1-decanol-based alcohol extraction method developed provides both high yield and selectivity while minimizing the environmental impact of the solvent used.

In the present study, Rhodamine 6G was successfully separated and preconcentrated from complex matrices, including food, beverages, cosmetics, hygiene products, and natural water, using an alcohol-based solvent extraction process created in accordance with green chemistry principles.

## 2. Materials and methods

### 2.1. Materials

In all the experimental investigations, analytically pure chemicals were employed. Every phase involved the use of deionized water (18 MΩ cm^−1^ (at 25 °C), conductivity, Millipore Milli-Q system, USA). In order to create diluted solutions from stock solutions (Sigma, Aldrich, St. Louis, MO, USA), Rhodamine 6G and matrix solutions (tartrazine, sunset yellow, azorubine, brilliant black, and lissamine green) were made with 100 mg L^−1^ ethanol and deionized water. The following alcohols were utilized straight from the stock solution (undiluted) when the method was being developed: 1-decanol (≥98%, 0.83 g mL^−1^), 1-pentanol (≥99%, 0.80 g mL^−1^), and 1-butanol (≥98.0%, 0.83 g mL^−1^). Ammonium (NH_4_^+^/NH_3_), (NaCH_3_COO-/CH_3_COOH) acetate, and (Na_2_HPO_4_^−^/NaH_2_PO_4_^2−^) phosphate buffer solutions with pH values between 2.0 and 9.0 were made.

### 2.2. Equipment

Rhodamine 6G was determined using a Hitachi model (UH-5300) UV–Vis double beam spectrophotometer (Tokyo, Japan) with a 10-mm optical path length. A WTW 3110 pH meter (WTW, Germany) was utilized. The compounds were weighed using a Radwag AS220/C/2 analytical balance (Radwag Balances & Scales, Poland) with a sensitivity of 0.1 mg. Ultrapure water was used. A Milli-Q system (Millipore, USA) device (18.2 MΩ cm^−1^, conductivity) was used to obtain distilled water. For experimental use, Nichipet EXII Volac brand micropipettes, adjustable between 100 and 1000 μL and 10 and 50 μL, were used. For the sonication effect to mix the model solutions homogeneously, an ultrasonic bath device from Weightlab Instruments, Sonorex (Model No. DT-255, Türkiye) was used.

HPLC-FLD was performed using a C18 column (2.7 μm, 4.6 × 150 mm). For each sample, 1.0 g of lipstick was precisely weighed into Falcon tubes. Cosmetic samples were dissolved in 20 mL of ethanol, whereas food samples were extracted with 20 mL of distilled water. Next, 100–500 μL of the resulting solution was taken, and the samples were subjected to alcohol-based solvent extraction before analysis. These samples were subjected to the LLME procedure before being injected into the HPLC system.

### 2.3. Test procedure

**Figure 1:**
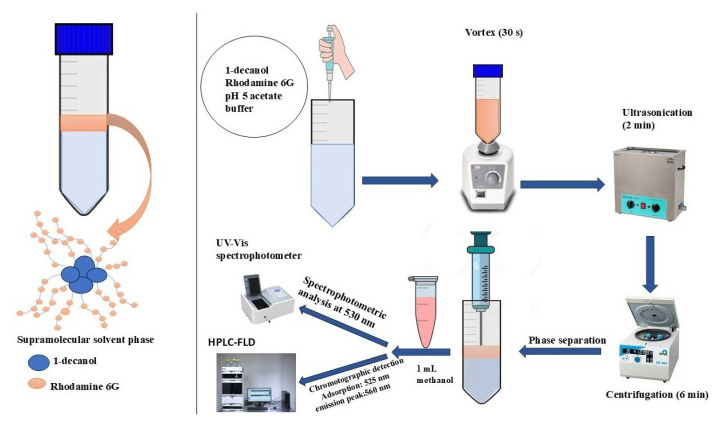
Schematic diagram of the procedure presented.

A 50 mL polypropylene conical tube was used to prepare the model solution. To this solution were added 100 μL of 1-decanol, 2 mL of phosphate buffer at pH 5, 100 μL of Rhodamine 6G at a concentration of 10 μg mL^−1^, and 10 mL of distilled water. The model solutions prepared for micelle formation were vortexed for 30 s and then sonicated in an ultrasonic bath for 60 s. The mixture was centrifuged at 4100 rpm for 6 min to separate the alcohol phase formed. After centrifugation, the aqueous phase was discarded and 1 mL of methanol was added to the 1-decanol phase containing Rhodamine 6G. UV–Vis spectrophotometry was used to measure the absorbance at the maximum wavelength (λmax) of 530 nm (Figure 1).

### 2.4. Real sample application

Real food, beverage, and cosmetic samples (cologne, powdered beverage, lipstick, liquid soap, water samples, confections, perfume, nail polish, chewing gum, and various beverages) were examined under ideal settings in order to assess the applicability of the method devised. One gram of lipstick was weighed and dissolved in 20 mL of ethanol to create the real samples. Twenty milliliters of distilled water was used to dissolve 1 g of chewing gum, sugar, and powdered drinks.

Between 100 and 500 μL of liquid samples, including cologne, liquid soap, perfume, and water samples, were collected.

## 3. Results and discussion

### 3.1. Optimization

#### 3.1.1. Effect of pH

**Figure 2:**
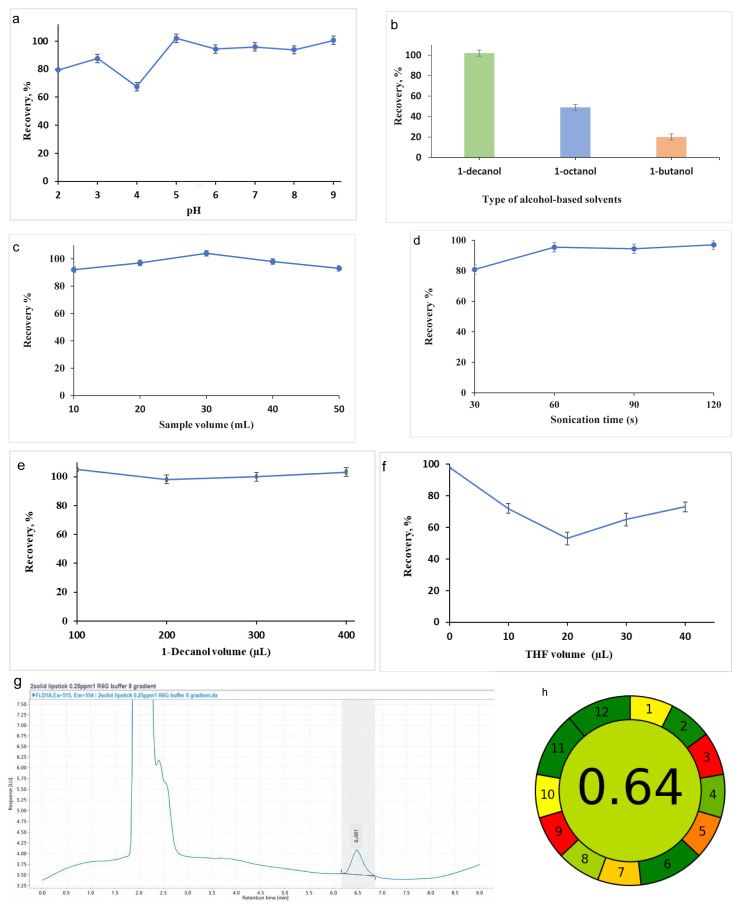
a) Recovery of Rhodamine 6G at different pH values (N = 3); 1-decanol volume: 100 μL, 2 mL pH 5 buffer solution, vortex time 30 s, sonication time 60 s, centrifugation 6 min, sample volume 10 mL (N = 3). b) Recovery of Rhodamine 6G with different alcohol species (N = 3); 1-decanol, 1-octanol, 1-butanol volume 100 μL, 2 mL of pH 5 buffer solution, vortex time 30 s, sonication time 60 s, centrifuge 6 min, sample volume 10 mL. c) Effect of sample volume on the recovery of Rhodamine 6G; 1-decanol volume 100 μL, 2 mL of pH 5 buffer solution, vortex time 30 s, sonication time 60 s, centrifugation 6 min (N = 3). d) Recovery of Rhodamine 6G with different sonication times; 1-decanol 100 μL, 2 mL of pH 5 buffer solution, vortex time 30 s, sonication time 30, 60, 90, 120 s, centrifugation 6 min, sample volume: 10 mL (N = 3). e) Recovery of Rhodamine 6G with different 1-decanol amounts (N = 3); volume of 1-decanol: 100–400 μL, 2 mL of pH 5 buffer solution, vortex time 30 s, sonication time 60 s, centrifuge 6 min, sample volume 10 mL. f) Effect of THF volume on the recovery of Rhodamine 6G (N = 3); 1-decanol volume: 100 μL, THF volume 0–40 μL, 2 mL of pH 5 buffer solution, vortex time 30 s, sonication time 60 s, centrifuge 6 min, sample volume 10 mL. g) Chromatography of the lipstick sample (530 nm). h) AGREEprep analytical greenness metric for sample preparation.

The concentration of positive charged ions rises as the pH medium gets more acidic, and the surfactant hydrophilic component lowers the pH activity in the solution medium. For ionic and uncharged molecules to interact, the pH medium must be regulated; the effect of this varies according to the type of surfactant. For separation–enrichment processes, pH is much more crucial due to the physical and chemical characteristics of dyes [51]. To find the optimum pH, alcohol-based solvent solution was created by adding 100 mL of 1-decanol and Rhodamine 6G to model solutions (2 mL of buffer solution) made between pH 2 and 9. Model solutions made with acetate buffer between pH 5 and 6 and with phosphate buffer between pH 2 and 4 and pH 7 and 9 were subjected to alcohol-based solvent extraction. When the elution solutions were evaluated by UV–Vis spectrophotometer at 530 nm following extraction, more stable and measurable recovery of Rhodamine 6G (>99%) was observed at pH 5 (Figure 2a). In the subsequent parameters, the pH of the model solution was adjusted to 5 (CH_3_COONa.3H_2_O with CH_3_COOH) using acetate buffer.

#### 3.1.2. Effect of alcohol types

1-Butanol (C4), 1-octanol (C8), and 1-decanol (C10) are examples of short-, medium-, and long-chain alcohols. The alcohol-based solution operation is depicted in Figure 2b. The recovery utilizing short-chain (recovery: 20%) or medium-chain (recovery: 49%) alcohols did not produce the intended outcome, as the figure illustrates. By employing 1-decanol (recovery, 102%), a long-chain alcohol, which forms micelles and extracts the Rhodamine 6G analyte into the organic phase, liquid–liquid microextraction of the analyte into the organic phase was facilitated. 1-Decanol has low water miscibility and easily separates into an organic phase, which makes phase separation easier and improves extraction efficiency during the microextraction procedure. As a result, during micelle production, 1-decanol enhances alcohol point performance.

#### 3.1.3. Sample volume

Sample volume is used to calculate the enrichment factor (EF), an analytical performance measure. Analysis was done on model solution volumes in model solution medium that ranged from 10 to 50 mL (Figure 2c). It was established that all the tested volumes resulted in quantitative recovery (≥ 95%). The volume of 50 mL was used for subsequent investigations.

#### 3.1.4. Effects of ultrasonication time

Ultrasonication time is a key parameter for quantitative analyte recovery in extraction studies. Following the addition of the alcohol-based solvent, the model solution was placed in an ultrasonic water bath to facilitate reverse micelle formation. The effect of ultrasonication time was evaluated in the range of 30 to 120 s. As shown in Figure 2d, an ultrasonication time of 60 s was determined to be sufficient for quantitative recovery.

#### 3.1.5. Effects of the amount of 1-decanol

To determine the optimal volume of 1-decanol for the quantitative recovery of the analyte, various amounts ranging from 100 to 400 μL were evaluated. As shown in Figure 2e, 100 μL of 1-decanol was sufficient to achieve quantitative recovery and was therefore selected for use throughout the study.

#### 3.1.6. Effects of tetrahydrofuran (THF) volume

This approach was used to examine the impact of THF quantity and volume. Analysis of recovery was done between 0 and 40 μL (Figure 2f). In order to adhere to the principles of green chemistry, the impact of THF on extraction efficiency was also examined during the optimization study. The findings showed that THF considerably reduced Rhodamine 6G recovery. This decline could be explained by THF’s potent solvation capacity, which can make the analyte more soluble in the aqueous phase and less likely to partition into the extraction phase. THF was therefore left out of the ideal extraction conditions. By avoiding the need for extra organic solvents, this finding further enhances the method’s ecofriendliness.

#### 3.1.7. Matrix effects

To investigate the matrix effect on the proposed LLME method, each matrix type was incorporated into the model solution. The recovery of the Rhodamine 6G analyte was evaluated under 10 different matrix conditions using quantitative analysis. The percentage recovery data for Rhodamine 6G in various matrices are presented in Table 1.

#### 3.1.8. Analytical performance

Using the proposed liquid microextraction method, a calibration graph (m, from which the slope value is calculated) was created at increasing analyte concentrations and applied to model solutions (blank solution) that did not include Rhodamine 6G. The analytical method performance, including correlation coefficients (R^2^), limit of detection (LOD), limit of quantification (LOQ), mean Rhodamine 6G recovery (R, %), and EF, were then specified. It was classified as LOD = (3 × σ)/(m × EF) and LOQ = (10 × σ)/(m × EF). The EF value was determined by dividing the maximum sample volume (50 mL) by the eluent (methanol) volume (1 mL) and was determined by dividing the standard deviation (SD) of the replicate appearance by its mean (M) and multiplying by 100 for the RSD% value. The RSD% value is calculated as (SD/M) × 100. The RSD% value was calculated by dividing the standard deviation (SD) of the replicate appearance by its mean and multiplying by 100. The RSD% is calculated as (SD/M) × 100.

#### 3.1.9. HPLC-FLD analysis

To validate the results with real sample applications obtained from the spectrophotometer, an Agilent 1260 Infinity II high-performance liquid chromatography-fluorescence detector system was used. Excitation and emission wavelengths for Rhodamine 6G were performed at 515 and 554 nm, respectively. The column used for the separation processes was an Agilent Poroshell 120 EC-C18 HPLC column (150 × 4.6 mm, 2.7 μm). The injection volume was 20 μL and the column temperature was set at 30 °C. The separation was carried out with an isocratic mobile phase system consisting of 55% ACN and 45% pH 5.0 phosphate buffer. The chromatogram data obtained were integrated using LC Openlab HPLC Agilent 1260 Infinity II software. The chromatogram of the lipstick sample is given in Figure 2g.

#### 3.1.10. Greenness assessment

The AGREE preparation tool, a practical software tool for utilizing greenness methodology, was first released in 2022. This tool focused on sample preparation in research. It was based on 12 impact categories and recalculated into 0–1 change subscores. These subscores were then used to program the assessment score. The assessment criteria included, among others, the selection and use of solvents, components, and reagents; waste generation; energy consumption; and their size and yield (Figure 2h) [52–56].

#### 3.1.11. Applications

In a model solution medium, the new alcohol-based solvent extraction technique was applied to slime, cologne, powdered beverage, lipstick, liquid soap, spray candy, perfume, water, lollipop, and pink soda samples. All real samples had quantitative recoveries ranging from 90% to 105% after application of the procedure under optimum conditions. Tables 2–4 show the addition–recovery results for Rhodamine 6G analyte at various concentrations (zero) and spiked (0.5–1.5 μg/g) for these real samples. Extraction studies developed with Rhodamine 6G are shown in Table 5.

In the present study, an alcohol-based solvent extraction method was developed for the determination of Rhodamine 6G in various matrices. Analyses were performed on samples of cosmetic products, food, and beverages to evaluate the applicability and reliability of the method. Alcohol-based solvent extraction was selected due to its advantages such as low solvent consumption, high enrichment factor, and short analysis time. After extraction, samples were analyzed using UV–Vis spectrophotometry. Additional measurements were performed using HPLC-FLD to confirm the results. The results showed that the method developed is an effective and reliable separation–preconcentration technique for the determination of Rhodamine 6G in complex matrices. Extraction efficiencies were compared among different sample types and matrix effects were evaluated (Table 5). Table 6 shows a comparison of the extraction method developed with separation and enrichment studies of Rhodamine 6G analyte in the literature.

## 4. Conclusions

During the present work, UV–Vis spectrophotometry was used to determine the amount of Rhodamine 6G in food, drink, and cosmetic goods utilizing a liquid–liquid phase microextraction technique based on alcohol-based solvents and dye-enriched Rhodamine 6G. The suggested method has a low detection limit, quantitative recovery results, good selectivity, and a high enrichment factor to identify Rhodamine 6G in food, beverage, and cosmetic products. Micelle formation was achieved without the use of THF, in accordance with green chemistry principles. Enrichment of Rhodamine 6G by separation using alcohol-based solvents eliminates the use of carcinogenic THF and offers significant advantages in terms of environmental sustainability and economics. Since the model solution only needs to be extracted once using an aqueous solution containing 0.1 mL of Rhodamine 6G, 2 mL of pH 5 phosphate buffer, and 0.1 mL of 1-decanol at a concentration of 10 μg mL^−1^, the suggested extraction process is quicker and simpler in comparison. Furthermore, solvent evaporation and cleaning are not required. As a result, the extraction procedure significantly increases the sample yield and takes roughly 20 min overall. Because it drastically lowers the consumption of organic solvents, it is also a low-cost separation–enrichment approach that can be employed in routine laboratory analysis without the need for additional equipment investment. The suggested alcohol-based microextraction method has a number of useful improvements over previously documented extraction techniques for Rhodamine 6G. The technique maintains acceptable analytical performance while offering a comparatively easy and quick extraction process with less solvent use. The current approach enables effective analyte recovery with little operational complexity, in contrast to many traditional extraction strategies that call for more complicated sample preparation procedures or higher quantities of organic solvents. These features highlight the suggested method’s suitability for regular analytical monitoring and make it a workable and ecofriendly substitute for determining Rhodamine 6G in food, cosmetic, and beverage samples.

## Figures and Tables

**Table 1:** Matrix effects on the recovery of Rhodamine 6G’s interactions with foreign ions (N = 3).

Matrix components	Added as	Concentration (mg/L)	Recovery (%)
Cl^−^	KCl	2500	105±3
K^+^	KNO_3_	5000	101±3
NO^−^_3_	NaNO_3_	2500	104±3
SO^2−^_4_	K_2_SO_4_	2500	102±3
Na^+^	NaNO_3_	5000	97±3
Sunset yellow		0.2	98±3
Azorubine		0.2	92±3
Tartrazine		0.2	105±3
Brilliant black		0.1	103±3
Lissamine green		0.1	102±3

**Table 2:** Study of Rhodamine 6G in water samples (N = 3).

Water sample	Added (mg/L)	Found(mg/L)	Recovery, %
Ankara tap water	0	BDL a	-
	0.5	0.48±0.03^b^	96±1
	1.0	1.06±0.05	100±2
	1.5	1.50±0.08	100±2
Incesu tap water	0	BDL	-
	0.5	0.50±0.02	100±1
	1.0	0.98±0.08	98±2
	1.5	1.55±0.11	103±2

a below detection limit

b Mean ± standard deviation

**Table 3:** Study of Rhodamine 6G in environmental cosmetic samples.

Cosmetic SAMPLE	Added (μg/g)	Found (μg/g)	Recovery, %
Orange slime	0	1.05±0.07^a^	-
	1.0	2.03±0.12	98±2
	1.5	2.60±0.14	103±2
Pink slime	0	0.77±0.05	-
	1.0	1.70±0.09	93±1
Liquid soap orange	0	BDL^b^	-
	1.0	1.0±0.07	100±3
	1.5	1.45±0.10	96±2
Liquid soap pink	0	BDL	-
	1.0	0.95±0.08	95±2
	1.5	1.45±0.10	96±2
Perfume	0	BDL	-
	1.0	1.0±0.07	100±1
	1.5	1.45±0.11	96±2
Nail varnish	0	0.10±0.01	-
	0.5	0.65±0.04	100±2
	1.5	1.65±0.09	103±2
Lipstick	0	BDL	-
	1.0	0.98±0.08	98±1
	1.5	1.45±0.09	96±3

a Mean ± standard deviation

b below detection limit

**Table 4:** Study of Rhodamine 6G in food and drink samples.

FOOD/DRINK SAMPLE	Added (μg/g)	Found (μg/g)	Recovery, %
Powdered drink	0	BDL^a^	-
	0.5	0.50±0.02^b^	100±2
	1.5	1.62±0.08	100±1
Powdered drink II	0	BDL	-
	1.0	1.02±0.11	102±2
	1.5	1.50±0.12	100±3
Powdered drink III	0	BDL	-
	1.0	0.97±0.06	100±1
	1.5	1.72±0.11	96±2
Pink soda	0	BDL	-
	0.5	0.50±0.04	100±1
	1.0	0.95±0.06	95±2
	1.5	1.45±0.10	96±2
Pink gum	0	BDL	-
	0.5	0.48±0.03	96±2
	1.0	0.95±0.08	95±2
	1.5	1.60±0.11	106±1
Orange gum	0	0.14±0.02	-
	0.5	0.64±0.05	100±2
	1.5	1.70±0.10	103±2
Yellow lollipop	0	BDL	-
	0.5	0.48±0.05	96±2
	1.0	0.97±0.08	97±2

a below detection limit

b Mean ± standard deviation

**Table 5:** HPLC-FLD results for addition–recovery for Rhodamine 6G.

Sample	Added, μg L^−1^	Found, μg L^−1^	Recovery %
**Tap water**	0	BDL^a^	-
	0.10	0.09±0.002^b^	94±1
	0.25	0.24±0.005	96±2
	0.5	0.48±0.007	96±2
**Powdered drink**	0	BDL	-
	0.10	0.09±0.006	97±2
	0.25	0.24±0.009	96±1
**Lipstick**	**Added, μg g** **^−1^**	**Found, μg g** **^−1^**	**Recovery %**
	0	0.07±0.004	-
	0.25	0.30±0.005	94±1
	0.50	0.55±0.008	96±1
**Colored gum**	**Added, μg g** **^−1^**	**Found, μg g** **^−1^**	**Recovery %**
	0	0.05±0.003	-
	0.25	0.28±0.004	92±2
	0.50	0.52±0.007	94±1

a below detection limit

b Mean ± standard deviation

**Table 6:** Comparison of the proposed extraction method with separation–preconcentration studies of Rhodamine 6G analyte found in the literature.

Methods	Extractant	LOQ ^a^	Recovery, %	Detection method	%RSD	Sample	Ref.
Hollow fiber liquid phase microextraction (HF-LPME)	1-octanole	10.0	%98.0	HPLC-DAD	%3.5	Waters samples, soft drinks, and lipsticks	[10]
Dispersive liquid-liquid microextraction DLLME	CHCl_3_ (chloroform)	7.97 ng mL^−1^	%83.0	UV-vis spectrophotometer	%2.88 and %1.47	Textile and plastic industry wastewater	[5]
Dispersive liquid-liquid microextraction DLLME	Trichloroacetic acid-trichloroacetate ethanol, chloroform	--	%88.1–%111.6	HPLC-FLD and UV-vis spectrophotometer	<4.4% and <4.7	Black tea, red wine, and hot pepper	[7]
Single-drop microextraction and optical probe fluorescence detection (DI-SDME-OP-FL)	Amyl acetate	0.50 nmol L^−1^	%93.2–103.2	UV-Vis spectrophotometer	%5.0–%10.5	Tap water, river water	[16]
Magnetic stirring assisted dispersive liquid-liquid microextraction (MSA-DLLME)	1-Octanol	1.23 ng mL^−1^	%100–%97	HPLC-DAD	%0.94	Environmental waters, beverages, and cosmetics	[8]
Emulsion liquid membrane (ELM	SPAN 80	-	%84	UV-Vis spectrophotometer	--	Wastewater	[4]
Alcohol-based solvent extraction	1-decanol	23.0 ng ml^−1^	%90–%106	UV-Vis spectrophotometer and HPLC-FLD	%1.01	Food, cosmetics and water samples	This work

LOQa: limit of quantification; EFb: enhancement factor; PFc: preconcentration factor; %RSD: relative standard deviation limit of quantification HPLC-FLD: high-performance liquid chromatography with fluorescence detection.

## Footnotes

1 Vaezi N, Karimi A, Dalali N. ZIF-8 as an adsorbent for the removal of Allura Red from aqueous solutions before determination by UV-vis spectrophotometry. Journal of Dispersion Science and Technology 2026, in press. https://doi.org/10.1080/01932691.2026.2635441

2 Sajjad S, Karshiev K, Tillayev S, Soylak M. Ternary deep eutectic solvent-based microextraction of rhodamine B from environmental matrices before UV–Vis analysis. International Journal of Environmental Analytical Chemistry 2026; in press. https://doi.org/10.1080/03067319.2026.2627619

## References

[1] Özkantar N Soylak M Tüzen M Spectrophotometric detection of rhodamine B in tap water, lipstick, rouge, and nail polish samples after supramolecular solvent microextraction Turkish Journal of Chemistry 2017 41 6 987 994 10.3906/kim-1702-72

[2] Zhong F Wang P He Y Chen C Li H Preparation of stable and superior flux GO/LDH/PDA-based nanofiltration membranes through electrostatic self-assembly for dye purification Polymers for Advanced Technologies 2019 30 7 1644 1655 10.1002/pat.4595

[3] Bokhale NB Bomble SD Dalbhanjan RR Mahale DD Hinge SP Sonocatalytic and sonophotocatalytic degradation of rhodamine 6G containing wastewaters Ultrasonics Sonochemistry 2014 21 5 1797 1804 10.1016/j.ultsonch.2014.03.022 24726320

[4] Hawwas AH Hashem MA Ismail MA Khalifa ME Preparation and characterization of new quaternary ammonium salt of guanidine-modified cellulose for effective removal of some anionic dyes Applied Water Science 2026 16 100 10.1007/s13201-025-02742-5

[5] Othman N Yi OZ Zailani SN Zulkifli EZ Subramaniam S Extraction of Rhodamine 6G dye from liquid waste solution: Study on emulsion liquid membrane stability performance and recovery Separation Science and Technology 2013 48 8 1177 1183 10.1080/01496395.2012.731123

[6] Ghanta R Mondal R Kar S Chowdhury T Chattopadhyay T Facile synthesis of Fe_3_O_4_@ NGQDs nanocomposites as an efficient fluorescent sensors and photocatalysts for the detection and degradation of Rhodamine B and Methylene Blue Microchemical Journal 2026 224 117557 10.1016/j.microc.2026.117557

[7] Saadat M Yamini Y Three-dimensional porous ZnCo-LDH/ZnCo-NH hybrid structure for electro-enhanced thin film microextraction of some acidic red dyes from various cosmetic and food samples Microchemical Journal 2026 223 117371 10.1016/j.microc.2026.117371

[8] Srivastava SK Pratap R Yadav M Singh S Chaudhary S Metal-free, dual-mode colorimetric and fluorometric sensing of Rhodamine-B using multifaceted biogenic carbon quantum dots: a biocompatible probe for biological and plant confocal imaging Luminescence 2026 41 2 e70441 10.1002/bio.70441 41677225

[9] Biparva P Ranjbari E Hadjmohammadi MR Application of dispersive liquid–liquid microextraction and spectrophotometric detection to the rapid determination of rhodamine 6G in industrial effluents Analytica Chimica Acta 2010 674 2 206 210 10.1016/j.aca.2010.06.024 20678631

[10] Chao Y Pang J Bai Y Wu P Luo J Graphene-like BN@ SiO_2_ nanocomposites as efficient sorbents for solid-phase extraction of Rhodamine B and Rhodamine 6G from food samples Food Chemistry 2020 320 126666 10.1016/j.foodchem.2020.126666 32229400

[11] Xiao N Deng J Huang K Ju S Hu C Application of derivative and derivative ratio spectrophotometry to simultaneous trace determination of rhodamine B and rhodamine 6G after dispersive liquid–liquid microextraction Spectrochimica Acta Part A: Molecular and Biomolecular Spectroscopy 2014 128 312 318 10.1016/j.saa.2014.02.180 24691361

[12] Ranjbari E Hadjmohammadi MR Optimization of magnetic stirring assisted dispersive liquid–liquid microextraction of rhodamine B and rhodamine 6G by response surface methodology: application in water samples, soft drink, and cosmetic products Talanta 2015 139 216 225 10.1016/j.talanta.2015.02.051 25882429

[13] Alruwaili G Ali HM Elnasr TAS Hasanin TH Alsohaimi IH New method based on solid-phase extraction and ultra high-performance liquid chromatography-fluorescence detector for the trace level detection of safranin and rhodamine B dyes in kid’s candies Journal of Food Composition and Analysis 2025 107800 10.1016/j.jfca.2025.107800

[14] Badiee H Zanjanchi MA Zamani A Fashi A Hollow fiber liquid-phase microextraction based on the use of a rotating extraction cell: A green approach for trace determination of rhodamine 6G and methylene blue dyes Environmental Pollution 2019 255 113287 10.1016/j.envpol.2019.113287 31600705

[15] Bayram E Bişgin AT Selective and simultaneous type-V deep eutectic solvent-based microextraction for optional-eliminative spectrophotometric determination of erythrosine and rhodamine B in foodstuffs, pharmaceuticals, and industrial samples Microchemical Journal 2025 114556 10.1016/j.microc.2025.114556

[16] Erbas Z Soylak M Determination of Rhodamine B by UV–Vis spectrophotometry in cosmetics after microextraction by using heat-induced homogeneous liquid–liquid extraction method Journal of the Iranian Chemical Society 2022 19 9 3935 3942 10.1007/s13738-022-02579-8

[17] Naseri M Shirani M Semnani A Shabanian M Asadpour S Highly efficient ZnFe_2_O_4_ nanoparticles decorated with sulfonated melamine for ultra-fast adsorptive removal of tartrazine from foodstuffs Food Analytical Methods 2026 19 3 117 10.1007/s12161-025-02972-y

[18] Yigit S Tuzen M Soylak M Dogan M Supramolecular solvent microextraction of Sudan blue II in environmental samples prior to its spectrophotometric determination International Journal of Environmental Analytical Chemistry 2016 96 6 568 575 10.1080/03067319.2016.1172221

[19] Bagheri Z Hashemi SH Keikha AJ Kaykhaii M Supramolecular solvent-based salt-saturated vortex microextraction coupled with UV–Vis spectrophotometry for sensitive determination of quercetin in food matrices Food Analytical Methods 2026 19 3 122 10.1007/s12161-026-03042-7

[20] Jagirani MS Soylak M Supramolecular solvents: a review of a modern innovation in liquid-phase microextraction technique Turkish Journal of Chemistry 2021 45 6 1651 1677 10.3906/kim-2110-15 38144606 PMC10734767

[21] Soylak M Celik M Uzcan F Supramolecular solvent-based microextraction of Sudan Orange G at trace levels for its separation, preconcentration and spectrophotometric determination International Journal of Environmental Analytical Chemistry 2020 100 8 935 944 10.1080/03067319.2019.1645842

[22] Skok A Vishnikin A Bazel Y Toth J Determination of Rhodamine 6G with direct immersion single-drop microextraction combined with an optical probe Plos one2024 2024 19 8 e0309121 10.1371/journal.pone.0309121 PMC11332950 39159159

[23] El-Ashtoukhy ESZ Fouad YO Liquid–liquid extraction of methylene blue dye from aqueous solutions using sodium dodecylbenzenesulfonate as an extractant Alexandria Engineering Journal 2015 54 1 77 81 10.1016/j.aej.2014.11.007

[24] Benkhedja H Ghouas H Benderrag A Haddou B Enhanced removal of toxic Disperse Blue 35 dye through cloud point extraction: influence of parameters and solvent regeneration Tenside Surfactants Detergents 2024 61 5 483 490

[25] Ghasemi E Kaykhaii M Application of a novel micro-cloud point extraction for preconcentration and spectrophotometric determination of azo dyes Journal of the Brazilian Chemical Society 2016 27 1521 1526 10.5935/0103-5053.20160030

[26] Shojaei S Shojaei S Nouri A Baharinikoo L Application of chemometrics for modeling and optimization of ultrasound-assisted dispersive liquid–liquid microextraction for the simultaneous determination of dyes NPJ Clean Water 2021 4 1 23 10.1038/s41545-021-00113-6

[27] Dil EA Ghaedi M Asfaram A Optimization and modeling of preconcentration and determination of dyes based on ultrasound assisted-dispersive liquid–liquid microextraction coupled with derivative spectrophotometry Ultrasonics sonochemistry 2017 34 27 36 10.1016/j.ultsonch.2016.05.013 27773245

[28] Narouei H Rahmani M Monitoring of curcumin in food samples using microextraction based on supramolecular solvent Journal of the Science of Food and Agriculture 2025 105 13 7382 7391 10.1002/jsfa.14442 40662591

[29] Unsal YE Soylak M Tuzen M Ultrasound-assisted ionic liquid-based dispersive liquid–liquid microextraction for preconcentration of patent blue V and its determination in food samples by UV–visible spectrophotometry Environmental monitoring and Assessment 2015 187 4 203 10.1007/s10661-015-4427-4 25800367

[30] Yıldız E Yaşar V Supramolecular solvent based microextraction with sugaring-out assisted liquid-liquid extraction for the determination of fungicides in honey Analytical Letters 2025 1 12 10.1080/00032719.2025.2478220

[31] Chen B Huang Y Dispersive liquid-phase microextraction with solidification of floating organic droplet coupled with high-performance liquid chromatography for the determination of Sudan dyes in foodstuffs and water samples Journal of Agricultural and Food Chemistry 2014 62 25 5818 5826 10.1021/jf5006403 24894629

[32] Nojavan S Tahmasebi Z Bidarmanesh T Behdad H Nasiri-Aghdam M Electrically enhanced liquid-phase microextraction of three textile azo dyes from wastewater and plant samples Journal of separation science 2013 36 19 3256 3263 10.1002/jssc.201300546 23894042

[33] Du RZ Zhang Y Bian Y Yang CY Feng XS Rhodamine and related substances in food: recent updates on pretreatment and analysis methods Food Chemistry 2024 459 140384 10.1016/j.foodchem.2024.140384 38996634

[34] Qi P Zeng T Wen Z Liang X Zhang X Interference-free simultaneous determination of Sudan dyes in chili foods using solid phase extraction coupled with HPLC–DAD Food Chemistry 2011 125 4 1462 1467 10.1016/j.foodchem.2010.10.059

[35] Prasad W Wani AD Khamrui K Hussain SA Khetra Y Green solvents, potential alternatives for petroleumbased products in food processing industries Cleaner Chemical Engineering 2022 3 100052 10.1016/j.clce.2022.100052

[36] Ozalp O Gumus ZP Soylak M Metal-organic framework functionalized with deep eutectic solvent for solid-phase extraction of Rhodamine 6G in water and cosmetic products Journal of Separation Science 2023 46 19 2300190 10.1002/jssc.202300190 37496320

[37] Fouda AA Abdallah AB Awad FS Green air-assisted LLME using a novel hydrophobic deep eutectic solvent for the extraction and quantification of Brilliant Green and Rhodamine B in food matrices Food Chemistry 2026 509 148465 10.1016/j.foodchem.2026.148465 41762880

[38] Li N Li Z Xu D Three novel Rhodamine 6G-based colorimetric and fluorescent pH switches Journal of Fluorescence 2025 35 2 1011 1023 10.1007/s10895-023-03574-9 38252215

[39] Guo J Han KS Mahurin SM Baker GA Hillesheim PC Rotational and translational dynamics of rhodamine 6G in a pyrrolidinium ionic liquid: a combined time-resolved fluorescence anisotropy decay and NMR study The Journal of Physical Chemistry B 2012 116 27 7883 7890 10.1021/jp303186v 22690897

[40] Wang Y Liu Y Han J Hu S Application of water-miscible alcohol-based aqueous two-phase systems for extraction of dyes Separation Science and technology 2011 46 8 1283 1288 10.1080/01496395.2010.551168

[41] Zohrabi P Shamsipur M Hashemi M Hashemi B Liquid-phase microextraction of organophosphorus pesticides using supramolecular solvent as a carrier for ferrofluid Talanta 2016 160 340 346 10.1016/j.talanta.2016.07.036 27591622

[42] Chen J Deng W Li X Wang X Xiao Y Hexafluoroisopropanol/Brij-35 based supramolecular solvent for liquid-phase microextraction of parabens in different matrix samples Journal of Chromatography A 2019 1591 33 43 10.1016/j.chroma.2019.01.030 30660441

[43] Soylak M Ahmed HEH Uzcan F Determination of Sudan III in food by supramolecular microextraction and spectrophotometry Analytical Letters 2023 56 6 997 1006 10.1080/00032719.2022.2112047

[44] Salamat Q Yamini Y Application of nanostructured supramolecular solvent based on C12mimBr ionic liquid surfactant to direct extraction of some chlorophenols in soil and rice samples Journal of Molecular Liquids 2022 366 120166 10.1016/j.molliq.2022.120166

[45] Altunay N Elik A Gürkan R Innovative and practical deep eutectic solvent based vortex assisted microextraction procedure for separation and preconcentration of low levels of arsenic and antimony from sample matrix prior to analysis by hydride generation-atomic absorption spectrometry Food Chemistry 2019 293 378 386 10.1016/j.foodchem.2019.05.019 31151625

[46] Alothman ZA Habila MA Yilmaz E Soylak M Alfadul SM Ultrasonic supramolecular microextration of nickel (II) as N, N′-Dihydroxy-1, 2-cyclohexanediimine chelates from water, tobacco and fertilizer samples before FAAS determination Journal of Molecular Liquids 2016 221 773 777 10.1016/j.molliq.2016.06.053

[47] Soylak M Agirbas M Yilmaz E A new strategy for the combination of supramolecular liquid phase microextraction and UV–Vis spectrophotometric determination for traces of maneb in food and water samples Food Chemistry 2021 338 128068 10.1016/j.foodchem.2020.128068 32950010

[48] Panda S Gorantla S Green analytical approaches and eco-friendly solvents: advancing industrial applications and environmental sustainability: a comprehensive review Oriental Journal of Chemistry 2025 41 2 10.13005/ojc/410231

[49] Ullah N Haseeb A Tuzen M Application of recently used green solvents in sample preparation techniques: A comprehensive review of existing trends, challenges, and future opportunities Critical Reviews in Analytical Chemistry 2024 54 8 2714 2733 10.1080/10408347.2023.2197495 37067946

[50] Ražić S Arsenijević J Mračević SĐ Mušović J Trtić-Petrović T Greener chemistry in analytical sciences: from green solvents to applications in complex matrices. Current challenges and future perspectives: a critical review Analyst 2023 148 14 3130 3152 10.1039/D3AN00498H 37337738

[51] Doğutan M Filik H Demirci S Apak R The use of palmitoyl hydroxyquinoline-functionalized Amberlite XAD-2 copolymer resin for the preconcentration and speciation analysis of gallium (III) Separation Science and Technology 2000 35 13 2083 2096 10.1081/SS-100102090

[52] Soylak M Sajjad S Salamat Q Ahmed HEH Polystyrene foam-based pipette-tip Micro-solid phase extraction (PFPT-μSPE) using Ni-MOF/S-CQDs nanocomposite: A novel method for efficient extraction of Lead from water and food samples Food Chemistry 2025 145101 10.1016/j.foodchem.2025.145101 40513487

[53] Wojnowski W Tobiszewski M Pena-Pereira F Psillakis E AGREEprep–analytical greenness metric for sample preparation TrAC Trends in Analytical Chemistry 2022 149 116553 10.1016/j.trac.2022.116553

[54] Pena-Pereira F Wojnowski W Tobiszewski M AGREE—Analytical GREEnness metric approach and software Analytical Chemistry 2020 92 14 10076 10082 10.1021/acs.analchem.0c01887 32538619 PMC7588019

[55] Dabi MM Alshareef FM Alwael H Abduljabbar TN Alkhraije AA Ultrasensitive miniaturized optical sensor for trace cyanide detection via supramolecular solvent microextraction of cyano-dithizone adduct Sensing and Bio-Sensing Research 2025 100836 10.1016/j.sbsr.2025.100836

[56] Hakami AAH Wabaidur SM Ali Khan M Alothman ZA Rafatullah M Siddiqui MR Development of ultra-performance liquid chromatography–mass spectrometry method for simultaneous determination of three cationic dyes in environmental samples Molecules 2020 25 19 4564 10.3390/molecules25194564 33036289 PMC7582281

